# Thermo-Coupler Thermometer

**Published:** 1915-11

**Authors:** 


**Thermo-Coupler Thermometer** for surgical coagulation procedures. Arthur F. Holding, N. Y., Med. Rec., Aug. 14. Haaland, Clowes, Lambert and Widal have shown that cancer and sarcoma cells are killed by a temperature of 50-55 c. for 20 minutes, whereas normal cells withstand temperature of 55-60. The methods of treating malignant tumors by coagulation are: Doyen, electric direct; Pfahler Electro-thermic; Nagelschmidt, Dessication, these three using D'Arsonval high frequency current of 1000-3000 milliamperes, bipolar. Clark Dessication, Oudin high frequency, 1 milliampere or less, monopolar; Percy, heat coagulation, the electric current being used merely to heat the cautery, illuminating current being used. Massey, Ionic galvanization, 500-1000 milliamperes. Beer, Dudin spark, misnamed fulguration, Oudin high frequency 1 milliampere or less, monopolar. Stroebel employs caustics en masse and Gelhorn acetone, to produce coagulation. The author describes at length, the principle and method of application of the thermo-couple, to measure the temperature employed and, by switches, a number of foci of determination can be established.

